# The Use of External Controls in FDA Regulatory Decision Making

**DOI:** 10.1007/s43441-021-00302-y

**Published:** 2021-05-20

**Authors:** Mahta Jahanshahi, Keith Gregg, Gillian Davis, Adora Ndu, Veronica Miller, Jerry Vockley, Cecile Ollivier, Tanja Franolic, Sharon Sakai

**Affiliations:** 1 BioMarin Pharmaceutical Inc, 105 Digital Drive, Novato, CA 94949 USA; 2 Forum for Collaborative Research, University of California School of Public Health, Washington, DC 20036 USA; 3 School of Medicine, UPMC Children’s Hospital of Pittsburgh, University of Pittsburgh, Pittsburgh, PA 15224 USA; 4 Aparito, Leiden Bioscience Park, BioPartner 3, 8 Galileiweg, 2333 BE Leiden, The Netherlands; 5 Tempest Therapeutics, 7000 Shoreline Court, Suite 275, South San Francisco, CA 94080 USA

**Keywords:** External controls, Retrospective natural history, Rare disease, Baseline controls, Historical controls

## Abstract

The regulatory standards of the United States Food and Drug Administration (FDA) require substantial evidence of effectiveness from adequate and well-controlled trials that typically use a valid comparison to an internal concurrent control. However, when it is not feasible or ethical to use an internal control, particularly in rare disease populations, relying on external controls may be acceptable. To better understand the use of external controls to support product development and approval, we reviewed FDA regulatory approval decisions between 2000 and 2019 for drug and biologic products to identify pivotal studies that leveraged external controls, with a focus on select therapeutic areas. Forty-five approvals were identified where FDA accepted external control data in their benefit/risk assessment; they did so for many reasons including the rare nature of the disease, ethical concerns regarding use of a placebo or no-treatment arm, the seriousness of the condition, and the high unmet medical need. Retrospective natural history data, including retrospective reviews of patient records, was the most common source of external control (44%). Other types of external control were baseline control (33%); published data (11%); and data from a previous clinical study (11%). To gain further insights, a comprehensive evaluation of selected approvals utilizing different types of external control is provided to highlight the variety of approaches used by sponsors and the challenges encountered in supporting product development and FDA decision making; particularly, the value and use of retrospective natural history in the development of products for rare diseases. Education on the use of external controls based on FDA regulatory precedent will allow for continued use and broader application of innovative approaches to clinical trial design, while avoiding delays in product development for rare diseases. Learnings from this review also highlight the need to update regulatory guidance to acknowledge the utility of external controls, particularly retrospective natural history data.

## Introduction

The United States Food and Drug Administration’s (FDA’s) drug approval standard requires *substantial evidence*^1^*of effectiveness* from *adequate and well*-*controlled investigations*^2^ including clinical investigations that incorporate, among other factors, a valid comparison to a control, to “distinguish the effect of a drug from other influences [1], such as spontaneous change in the course of the disease, placebo effect, or a biased observation” [1]. The FDA, consistent with regulations (21 CFR 314.126) and ICH E10 guidance, generally recognizes internally^3^ controlled [1] study designs (placebo, active treatment, dose comparison, no-treatment) where “the control group and test groups are chosen from the same population and treated concurrently” [1]. However, FDA does recognize that in studies for diseases with high and predictable mortality or progressive morbidity, and in particular for certain rare diseases, when it is not feasible or would not be considered ethical to use an “internal control”, reliance on “external controls”^4^,^5^ may be acceptable [1, 2]. When a trial is externally controlled, the results of treatment with the test drug may be compared with experience derived from the adequately documented natural history of the disease or condition, a registry, published literature, or patient medical records [3, 4]. Patients may also serve as their own controls [1] (by comparison to their status before therapy).^6^

In this article, we briefly review guidance documents discussing the use of external controls and provide examples of approvals where external controls were deemed satisfactory to meet FDA standards for approval. We highlight some methodological and statistical considerations and advocate for a change in guidance to promote the continued use of external controls, including retrospective natural history, in drug development and approval.

### Definitions and Categories of External Controls

The ICH E10 guidance defines an externally controlled trial as “one in which the control group consists of patients who are not of part of the randomized study as the group receiving the investigational agent i.e., there is no concurrently randomized control group” [1].

External controls can be categorized by the *time the subject data were collected into* [1, 3, 4]:

- *Concurrent External Controls:* The control group is based on subject level data *collected at the same time as the treatment arm but in another setting* [1]. An example is data from a concurrent prospective natural history^7^ study as the control arm for an open-label treatment study.
- *Non*-*concurrent External Controls (Historical Control):* The control group is based on data collected *at a time different (e.g., historical) from the treatment arm*. Such “historical controls” can be derived from several different types of sources including:
  - **Retrospectively Collected Natural History**: Subject level data collected retrospectively from a natural history study. Such data may be extracted from sources *such as existing medical records* (*for example patient charts* [3, 4])*, or from a previously conducted registry.*^8^
  - **Published Data**: Data only available in the *published literature*. Such published data may have been derived from individual cases, however, it is distinguished from retrospectively collected natural history data based on the lack of access to subject level data and the lack of detailed information on data collection methodology.
  - **Previous Clinical Study**: Subject level data from an arm of a previously completed clinical study [3] in the same indication and/or patient population.
  - **Baseline-Controlled Study**: Historical control derived from a patient’s baseline (“patient baseline control or baseline-controlled study”) [1]. The data could be collected over a period of time prior to initiation of treatment, and patients’ status on therapy is compared with status before therapy.

Of note, the term “real-world-evidence” (RWE)^9^ has recently been used to describe data sourced from natural history studies, chart reviews, registries and other settings and used as a comparison arm for a single-arm study [5–7].

### Summary of Current Guidance Discussing Use of External Controls

Several guidance documents discuss the use of external controls as a comparator in clinical trials [1–4, 8–14] (see Table 1), particularly for rare diseases. The ICH E10 guideline on “Choice of control group and related issues in clinical trials^”^ [1] provides a comprehensive discussion on such controls, stating “The choice of the control group should be considered in the context of available standard therapies, the adequacy of the evidence to support the chosen design, and ethical considerations.” While the guideline emphasizes that in most situations, an internal concurrent control is necessary to minimize bias and obtain robust statistical analyses, it also highlights that this may not always be feasible. In addition, it is broadly recognized by developers and researchers that initiation of prospective natural history studies for use as a source of external controls may not be feasible, especially in rare diseases, thus alternative approaches, such as the use of retrospective natural history data, have frequently been leveraged to support product development and approval. ICH E10 envisages this flexibility by acknowledging the acceptability of external controls from a group of patients treated at an earlier time (“historical”). Additionally, the FDA guidance “Rare diseases: common issues in drug development” [4] emphasizes that product development should not be delayed due to the lack of prospective natural history data. While FDA highlights the use of prospectively collected natural history data as the preferred approach, the guidance specifically, states that “initiation of prospective natural history studies should not delay interventional testing otherwise ready to commence for a serious disease with unmet medical need” [4]. This point abides by a pragmatic approach to product development and underscores the importance of ensuring development can proceed to expedite patient access to treatment.

**Table 1:** FDA and ICH regulatory guidance discussing the use of external controls

Type	Title	Date
Final draft	ICH E10: choice of control group and related issues in clinical trials—also published as an FDA final draft guidance dated May 2001	July 2000 & May 2001
Final	GfI^a^: use of Bayesian statistics in medical device clinical trials	Feb 2010
Final	GfI: expedited programs for serious conditions	May 2014
Final	GfI: duchenne muscular dystrophy and related dystrophinopathies: developing drugs for treatment	Feb 2018
Draft	Rare diseases: common issues in drug development (Revision 1)	Feb 2019
Final	GfI: expedited Programs for regenerative medicine therapies for serious conditions	Feb 2019
Draft	GfI: rare diseases—natural history studies for drug development	March 2019
Draft	GfI: interacting with the FDA on complex innovative trial designs for drugs and biological products	Sept 2019
Final	GfI: adaptive designs for clinical trials of drugs and biologics	Nov 2019
Draft	GfI: demonstrating substantial evidence of effectiveness for human drug and biological products	Dec 2019
Final	GfI: human gene therapy in rare diseases	Jan 2020

^a^Guidance for industry

FDA further articulates the importance of flexibility in trial design in the recently published FDA draft guidance on “Demonstrating substantial evidence of effectiveness for human drug and biological products” [2]. The draft guidance indicates that FDA may rely on study designs that produce less certainty (such as externally controlled studies) in some circumstances such as “life-threatening and severely debilitating diseases with an unmet medical need,^10^ for certain rare diseases, or potentially even for a more common disease where the availability of existing treatments makes certain design choices infeasible or unethical” [2]. The guidance also notes that a single trial with compelling results compared to either an external or concurrent control, could further be supported by data from separate sources (e.g., a natural history study, case report forms, or registries) as confirmatory evidence.

**Fig. 1:**
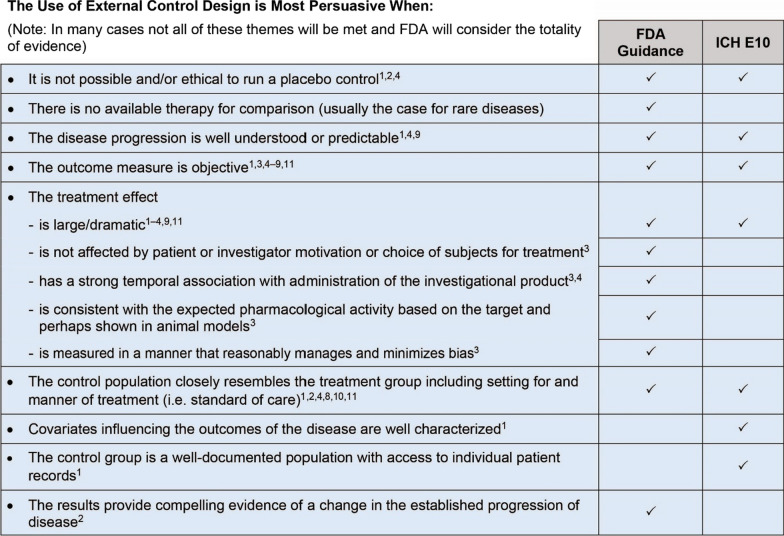
The use of external control design is most persuasive under the following circumstances

Collectively, these guidance documents [1–4, 8–14] reaffirm that use of external controls is acceptable under certain circumstances (see Fig. 1 for details) and reinforce the need for flexibility both in guidance as well as in application during product development and FDA decision making.

## Methods

We searched FDA regulatory approvals between 2000 and 2019^11^ for drug and biologic products where pivotal studies employed external controls. We included original marketing applications and supplemental applications for new indications specifically mentioning use of natural history data or historical controls to support a pivotal study. Applications in which natural history data were used in other ways, such as to guide endpoint development or to interpret nonclinical studies, were excluded.

Since the use of external controls appears to be well accepted in the field of oncology [15, 16], we focused our assessment on non-oncology product approvals, concentrating on the FDA divisions responsible for reviewing the following therapeutic areas: gastroenterology and inborn errors of metabolism; neurology; metabolism and endocrinology; reproduction, bone diseases, and urology; and non-malignant hematology. Anti-infectives, vaccines and immunoglobulins were excluded as their development pathways are dictated by guidelines unique to the therapeutic area.

We examined the characteristics of such applications with respect to rare disease status, seriousness of the disease, degree of unmet medical need, and objectivity of the primary endpoint and categorized the source of the external control data.

## Results

**Fig. 2:**
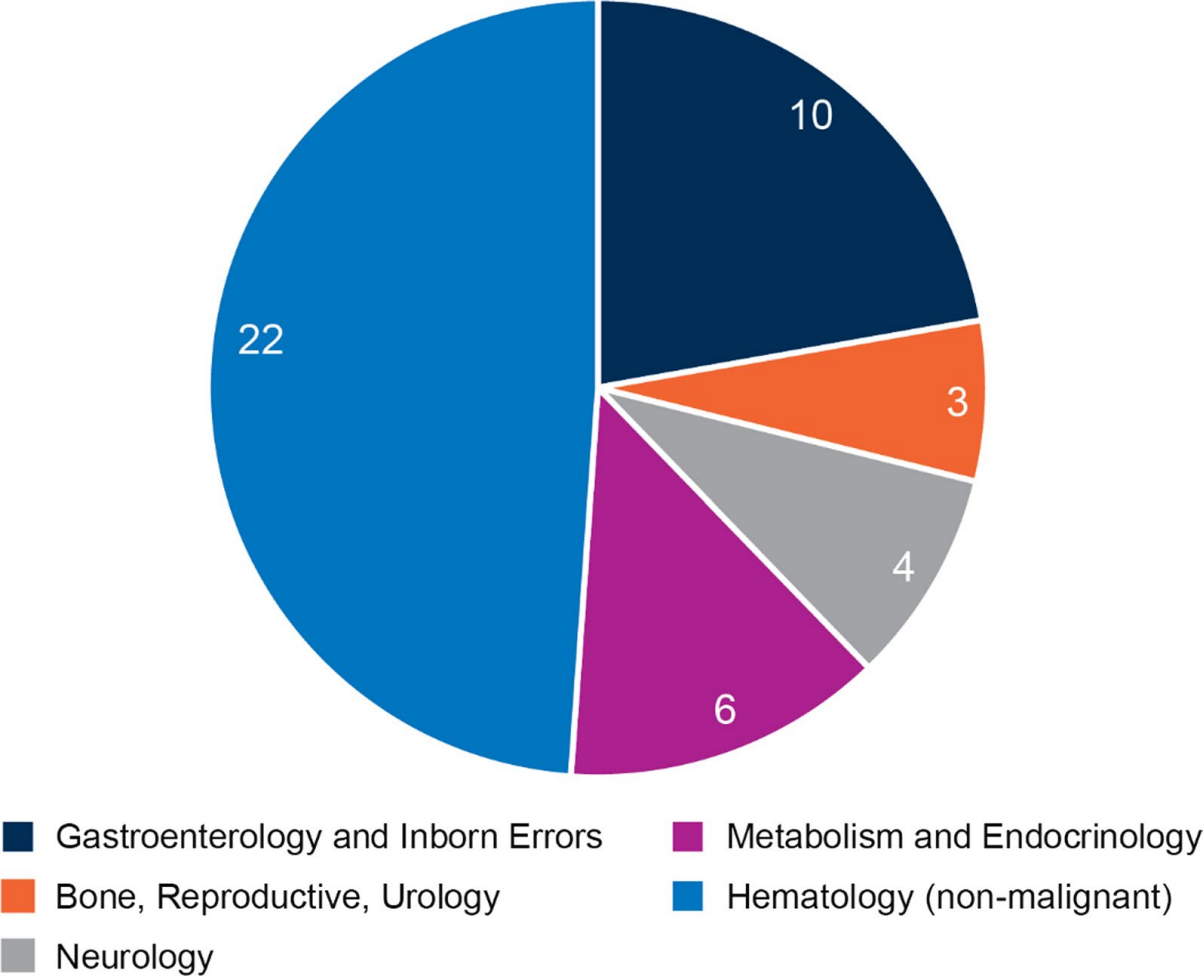
FDA review divisions responsible for the 45 product approvals relying on external controls (2000–2019, select therapeutic areas)

Based on our search criteria, we identified forty-five products^12^ (see Table 2) for which pivotal trials were supported by external controls. Nearly half (49%) of the cases identified were for non-malignant hematological products (Fig. 2) with gastroenterology and inborn errors of metabolism products comprising the second largest category (22%), followed by metabolism and endocrinology products (13%), neurology products (9%), and reproduction, bone diseases and urology products (7%), illustrating that experience with the use of external controls is variable across the Divisions at FDA.

**Table 2:** Characteristics of US FDA approvals based on external controls 2000–2019 (selected indications; *n* = 45)—presented in reverse order of approval

Product/approval date	Condition	Rare disease^a^	Unmet Need^b^	Objective endpoint?	Pivotal study(ies) design	**Source of External Control**
**Zolgensma** (onasemnogene abeparvovec-xioi) May 24, 2019	Spinal muscular atrophy	✓	✓	✓	Phase 1, open-label, single-arm, single center, ascending dose (*N* = 15; 3.4–6.3 months) Phase 3, open-label, single-arm (*N* = 21; 3.9 months)^c^	Retrospective natural history
**Esperoct** (recombinant antihemophilic factor) glycoPEGylated-exei Feb 19, 2019	Hemophilia A^d^	✓	✓	✓	Phase 3, open-label, non-randomized, two arms, multicenter (*N* = 175 subjects on prophylactic treatment; 12–66 y/o)^e^; 1 year (main phase)	Baseline control
**Omegaven** (fish oil triglyceride) July 17, 2018	Parenteral nutrition-associated cholestasis in pediatrics	✓	✓	✓	Phase 2–3, open-label (*N* = 52; < 2 y/o) Compassionate use, open-label (*N* = 30; < 5 y/o); median treatment duration: 2.7 months	Retrospective natural history
**Crysvita** (burozumab-twza) Apr 17, 2018	X-linked hypophosphatemia in pediatrics^f^	✓	✓	✗	Phase 2, open-label, randomized, multicenter, dosing interval dose titration (*N* = 52; 5–14 y/o); 64 weeks Phase 2, open-label, randomized, single-arm, multicenter (*N* = 13; 1–4 y/o); 24 weeks^g^	Retrospective natural history
**Brineura** (cerliponase alfa) April 27, 2017	Late infantile neuronal ceroid lipofuscinosis type 2	✓	✓	✗	Phase 1–2, open-label, non-randomized, single-arm, dose-escalation (*N* = 24; 3–8 y/o); 48 weeks with 96-week extension	Retrospective natural history
**Tepadina** (thiotepa) Jan 26, 2017	Graft rejection prior to HSCT^h^ in children with Class 3 beta-thalassemia	✓	✓	✓	Retrospective, observational, multicenter (*N* = 25; 5–16 y/o); Up to 1 year post-HSCT	Retrospective natural history
**Exondys 51** (eteplirsen) Sep 19, 2016	DMD amenable to Exon 51 skipping	✓	✓	✗	Phase 1–2, randomized, multi-dose, placebo-controlled (*N* = 12; 7–11 y/o); 24 weeks Open-label extension of the phase 1-2 study (*N* = 12); 212 weeks	Retrospective natural history
**Afstyla** (recombinant single chain analogue of factor VIII) May 25, 2016	Hemophilia A^d^	✓	✗	✓	Phase 1–3, open-label, multicenter, cross-over (*N* = 146 subjects on prophylactic treatment; ≥ 12 y/o); 8.5 months Phase 3, open-label, multicenter (*N* = 84; 0– < 12 y/o); 5.6 months	Baseline control
**ProvayBlue** (methylene blue) April 8, 2016	Acquired Methemoglobinemia	✓	✓	✓	Retrospective chart review of case series (*N* = 6; 6 days to 69 y/o) Series of cases from publications (*N* = 41; 9 days to 80 y/o)	Baseline control
**Defitelio** (defibrotide sodium) Mar 30, 2016	Hepatic veno-occlusive disease with renal or pulmonary dysfunction post-HSCT^h^	✓	✓	✓	Phase 3, open-label, multicenter study (*N* = 102; pediatrics and adults); 180 days post-HSCT	Retrospective natural history
**Idelvion** (recombinant fusion von Willebrand Factor) Mar 4, 2016	Hemophilia B^d^	✓	✗	✓	Phase 2–3, open-label, non-randomized, multicenter (*N* = 23 on-demand treatment switched to prophylactic treatment & *N* = 40 on prophylactic treatment; ≥ 12 y/o); Up to 27 months	Baseline control
**Kanuma** (sebelipase alfa) Dec 8, 2015	Lysosomal acid lipase deficiency in infants (Wolman disease)	✓	✓	✓	Phase 1–2, open-label, single-arm, dose-escalation (*N* = 9; 1–6 months)	Retrospective natural history
**Vonvendi** (recombinant von Willebrand Factor) Dec 8, 2015	von Willebrand disease in adults	✓	✓	✗	Phase 3, open-label, uncontrolled, multicenter, two dose levels (*N* = 22; ≥ 18 y/o); 12 months	Baseline control
**Strensiq** (asfotase alfa) Oct 23, 2015	Perinatal/ infantile- and juvenile-onset hypophosphatasia	✓	✓	✓	Two phase 2, open-label, single-arm, multicenter (Total *N* = 68); 48 weeks	Retrospective natural history
**Nuwiq** (recombinant antihemophilic factor) Sept 4, 2015	Hemophilia A^d^	✓	✗	✓	Phase 3, open-label, single-arm, uncontrolled, multicenter (*N* = 32, adults); ≥ 6 months Phase 3, open-label, single-arm, uncontrolled, multicenter (*N* = 56; 2–12 y/o); ≥ 6 months	Previously conducted clinical study
**Cholbam** (cholic acid) Mar 17, 2015	Bile acid synthesis disorders	✓	✓	✓	Open-label, non-randomized study and its extension (*N* = 44); 21 months Published case series (*N* = 15)	Baseline control
**Xuriden** (uridine triacetate) Sep 4, 2015	Hereditary orotic aciduria	✓	✓	✓	Open-label, single-arm, baseline-controlled (*N* = 4; 3– 19 y/o); 6 weeks with 6-month extension	Baseline control
**Myalept** (metreleptin) Feb 23, 2014	Congenital and acquired generalized lipodystrophy as adjunct to diet	✓	✓	✓	Open-label, single-arm, uncontrolled (*N* = 9; > 14 y/o); 1 year Open-label, single-arm, uncontrolled (*N* = 63; 1–14 y/o); 1 year	Baseline control
**Vimpat** (lacosamide) Aug 29, 2014^i^	Partial onset seizure (monotherapy)	✗	✓	✓	Randomized, multicenter, single-blind, two dose levels (*N* = 425; ≥ 16 y/o); 10 weeks (plus 6 weeks withdrawal of antiepileptic drugs)	Previously conducted clinical study
**Tretten** (recombinant coagulation Factor XIII A) Dec 23, 2013	Congenital Factor XIII A-subunit deficiency	✓	✓	✓	Phase 3, open-label, uncontrolled, multicenter (*N* = 41; ≥ 6 years); 12 months	Retrospective natural history
**Novoeight** (recombinant antihemophilic factor) Oct 15, 2013	Hemophilia A^d^	✓	✗	✓	Phase 3, open-label, single-arm, uncontrolled, multicenter (*N* = 150; ≥ 12 y/o); At least 75 days	Published data
**Rixubis** (recombinant Factor IX) June 26, 2013	Hemophilia B^d^	✓	✓	✓	Phase 1–3, Part 2: open-label, uncontrolled, multicenter (*N* = 70; ≥ 12 years); 56 subjects treated prophylactically for median duration of 6 months and 14 subjects treated on-demand for median duration of 3 months	Published data
**Octaplas** (plasma protein fraction) Jan 17, 2013	Replacement of multiple coagulation factors	✗	✓	✓	None were considered pivotal: Open-label, non-randomized, parallel group (*N* = 20) Phase 2, single-blind, randomized, controlled (*N* = 55) Open-label non-randomized (*N* = 36) Open-label, randomized (*N* = 60)	Previously conducted clinical study
**Juxtapid** (lomitapide) Dec 21, 2012	Homozygous familial hypercholesterolemia	✓	✓	✓	Open-label, single-arm (*N* = 29, 18–55 y/o); 26 weeks	Baseline control
**Signifor** (pasireotide diaspartate) Dec 14, 2012	Cushing disease in adults	✓	✓	✓	Phase 3, randomized, double-blind, multicenter, two-dose regimen (*N* = 162); 6 months	Baseline control
**Elelyso** (taliglucerase alfa) May 1, 2012	Type 1 Gaucher	✓	✓	✓	Phase 3, randomized, double-blind, parallel-dose group, multicenter (*N* = 31; 19–74 y/o - all patients were enzyme replacement therapy naïve); 9 months Phase 3, open-label, single-arm, multicenter (*N* = 25 patients switched from imiglucerase to Elelyso; 13–66 y/o); 9 months	Baseline control
**Ferriprox** (deferiprone) Oct 14, 2011	Transfusional iron overload due to thalassemia syndromes^j^	✓	✓	✓	Prospectively planned and retrospectively selected patients (in whom previous chelation therapy was inadequate) from pooled previously conducted clinical studies of different designs (*N* = 236; mean age 18.2 y/o); Up to 1 year	Baseline control
**Soliris** (eculizumab) Sept 23, 2011 ^k^	Atypical hemolytic uremic syndrome	✓	✓	✓	Phase 2, open-label, single-arm, multicenter (*N* = 16; 17–68 y/o); ≥ 26 weeks Phase 2, open-label, single-arm, multicenter (*N* = 20; 13–63 y/o); ≥ 26 weeks Retrospective, open-label, single-arm, multicenter (*N* = 30; nineteen 2 months to < 18 y/o, and eleven adults); ≥ 26 weeks	Baseline control
**Corifact** (Factor XIII concentrate) Feb 23, 2011	Prophylactic treatment of congenital Factor XIII deficiency	✓	✓	✓	Phase 2, open-label, single-arm (*N* = 14); 12 weeks	Published Data
**Lamictal XR** (lamotrigine) Apr 25, 2011 ^l^	Conversion to monotherapy in patients ≥ 13 years with partial seizures	✗	✓	✓	Phase 3, randomized, double-blind, two-dose levels (*N* = 223; ≥ 13 y/o); 12 weeks	Previously conducted clinical study
**Anascorp** (centruroides scorpion anti-venom) Aug 4, 2011	Clinical signs of scorpion envenomation	✓	✓	✗	Phase 3, randomized, placebo-controlled (*N* = 8 on drug; 7 on placebo; 1 month to 18.7 y/o) Supported by four phase 2-3, open-label studies (*N* = 1,526) using historical data (retrospective chart review; *N* = 97) as external control	Retrospective natural history
**Carbaglu** (carglumic acid) Mar 18, 2010	Hyperammonemia due to N-acetyl glutamate synthase (NAGS) deficiency	✓	✓	✓	Retrospective analysis of case histories of 23 patients (newborn to 13 y/o) treated with carglumic acid between 1991 and 2007	Retrospective natural history
**Vpriv** (velaglucerase alfa) Feb 26, 2010	Type 1 Gaucher	✓	✓	✓	Phase 3, randomized, double-blind, multicenter, parallel-dose group (*N* = 25; ≥ 4 y/o); 12 months Phase 3, randomized, double-blind, active-controlled (imiglucerase), parallel group, multicenter (*N* = 34; 17 received VPRIV; ≥ 3 y/o); 9 months Phase 3, open-label, single-arm, multicenter (*N* = 40; patients switched from imiglucerase to VPRIV; ≥ 9 y/o); 12 months	Baseline control
**Acthar** (corticotropin) Oct 15, 2010	Infantile spasm monotherapy	✓	✓	✓	Randomized, single-blind, active-controlled (*N* = 15 plus 14 on active)	Published data
**Atryn** (recombinant human anti-thrombin) Feb 6, 2009	Venous thromboembolism in surgery of patients with congenital antithrombin deficiency	✓	✓	✓	Phase 3, open-label Phase 2, open-label (Pooled patient data *N* = 31)	Retrospective natural history
**Ceprotin** (protein C concentrate) Mar 30, 2007	Venous thrombosis and purpura fulminans	✓	✓	✓	Phase 2–3, open-label, non-randomized (*N* = 18; newborn to 25.7 y/o)	Retrospective natural history
**Implanon** (etonogestrel) July 17, 2006	Prevention of pregnancy	✗	✓	✓	Four open-label studies (*N* = 1,117); 2–3 years	Published data
**Myozyme** (alglucosidase alfa) Apr 28, 2006	Infantile-onset Pompe	✓	✓	✓	Phase 2–3, randomized, open-label, multicenter, dose-ranging (*N* = 19); 52 weeks	Retrospective natural history
**Ammonul** (sodium phenylacetate and sodium benzoate) Feb 17, 2005	Hyperammonemia	✓	✓	✓	Retrospective analysis of patients (*N* = 316; newborn to 53 y/o) treated between 1981 and 2003 in an open-label compassionate use study	Retrospective natural history
**Orfadin** (nitisinone) Jan 18, 2002	Hereditary Tyrosinemia type 1	✓	✓	✓	Phase 2–3, open-label, uncontrolled, multicenter, compassionate use (*N* = 207; median age 9 months); 22 months (median duration of treatment)	Retrospective natural history
**Digifab** (ovine digoxin fab injection) Aug 31, 2001	Digoxin toxicity or overdose	✗	✓	✓	Open-label (*N* = 15) Pharmacokinetic/Pharmacodynamic study in HHVs^m^ (Digifab *N* = 8 versus Digibind *N* = 8)	Previously conducted clinical trial
**Venofer** (iron sucrose injection) Nov 6, 2000	Iron deficiency anemia	✗	✓	✓	Phase 2–3, open-label, multicenter, historical-controlled (*N* = 101); 10 weeks Open-label, multicenter, baseline-controlled (*N* = 23) Open-label, multicenter, baseline-controlled (*N* = 132)	Retrospective natural history
**Cetrotide** (cetrorelix acetate) Aug 11, 2000	Inhibition of premature LH^n^ surges in women undergoing controlled ovarian stimulation	✗	✓	✓	Phase 3, randomized, open-label, multicenter, active-controlled^o^ (*N* = 188 vs 86 on active); 1 to 19 days Phase 3, non-controlled, open-label, multicenter (*N* = 346); 1 to 15 days Phase 3, randomized, open-label, multicenter, active- controlled^o^ (*N* = 115 versus 39 on active), one dose	Retrospective natural history
**Argatroban** (argatroban) June 30, 2000	Thrombosis in patients with heparin-induced thrombocytopenia	✗	✓	✓	Open-label, non-randomized, multicenter (*N* = 309)	Retrospective natural history
**Hectorol** (doxercalciferol) April 6, 2000	Secondary hyperparathyroidism in patients undergoing chronic renal dialysis	✗	✓	✓	Phase 3, open-label, multicenter (*N* = 28; 23–73 y/o); 20 weeks including 8-week washout period Phase 3, open-label, multicenter (*N* = 42; 28–76 y/o); 20 weeks including 8-week washout period	Baseline control

^a^Rare disease does not necessarily mean the product has orphan drug designation

^b^Unmet medical need (no existing therapy, inadequate existing therapy, or better safety)

^c^Study was ongoing

^d^External control was used for the routine prophylaxis indication only

^e^Years old

^f^Only the pediatric indication relied on use of historical controls

^g^Study was ongoing and 24-week data were submitted in the BLA and 40-week primary analysis results were submitted during the BLA review process

^h^Hematopoietic stem-cell transplantation

^i^Efficacy supplement 013

^j^When chelation therapy is inadequate

^k^Efficacy supplement 172

^l^Efficacy supplement 006

^m^Healthy human volunteers

^n^Luteinizing hormone

^o^Active control was not approved in the US

### Overall Trends

The majority (80%; see Table 3) of the approvals relying on external controls were for a rare disease where regulatory flexibility was applied due to the size of the population and/or to the unmet medical need (i.e., no or inadequate available therapy). This is consistent with other reports of FDA flexibility with respect to the quantum of evidence relied upon for the approval of orphan products [15, 17]. Overall, for the therapeutic categories evaluated, approximately one in three (33%) first-time approvals of products for rare diseases relied on external controls over the 20-year period.

**Table 3:** Characteristics of products approved based upon use of external controls (2000–2019)

	US (*N* = 45)
Rare disease^a^	36 (80%)
Use of objective endpoint	39 (87%)

^a^These were products for rare diseases which did not necessarily have an orphan drug designation

### Types of External Controls Used to Support Approval

**Fig. 3:**
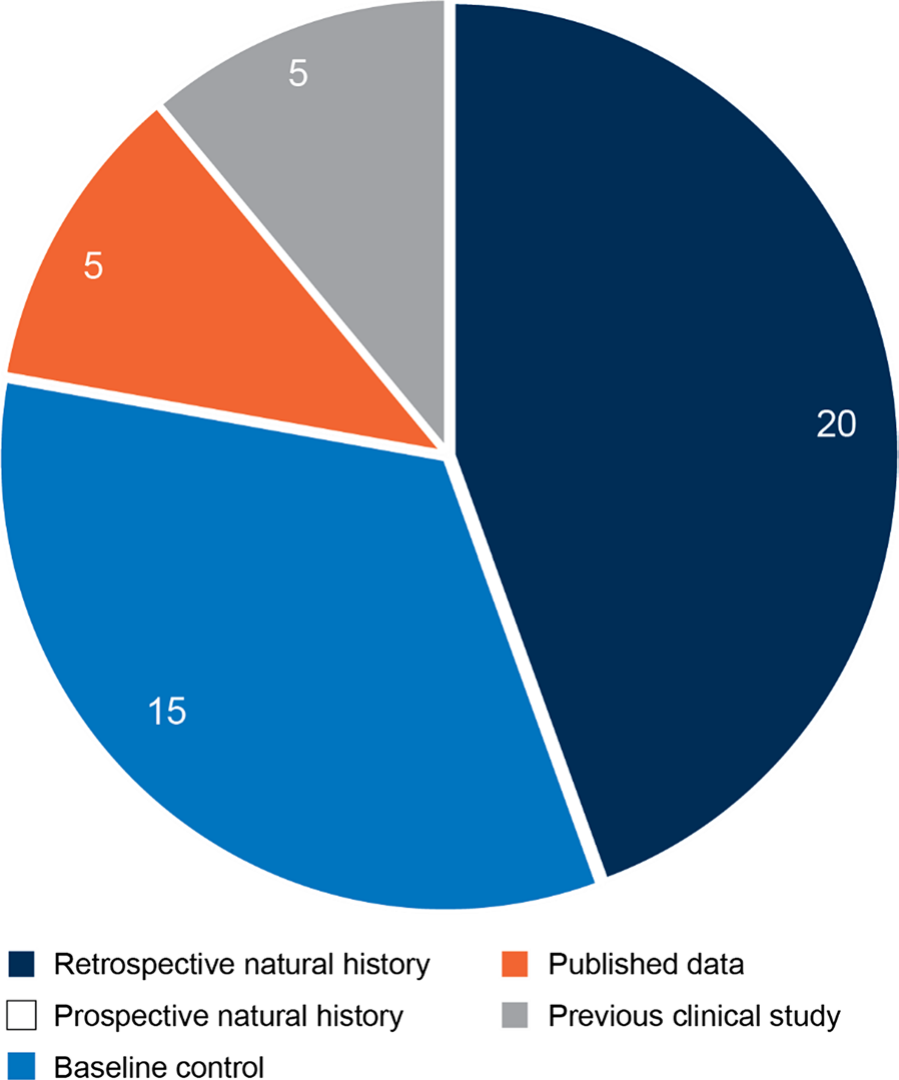
Categories of external controls to support product approval by the US FDA (2000–2019, select therapeutic areas)

Of the 45 approvals evaluated, historical controls derived retrospectively from natural history data were the most common source (44%) of external control, including retrospective reviews of patient medical records (see Fig. 3). While prospectively gathered natural history data sources are preferred based on FDA guidance, none of the external controls included in the regulatory approvals assessed in this review were prospective. Other data sources of external control were less common (baseline control: 33%; published data: 11%; data from a previous clinical study: 11%). A hybrid approach, where external control data were added to a concurrent randomized control arm (placebo and/or active), was used for at least three products (velaglucerase alfa, corticotropin, centruroides anti-venom) developed to treat conditions for which there were no available therapies. Two of these were approved for the treatment of rare pediatric conditions.

### Objective Versus Subjective Endpoints

The vast majority (87%) of cases identified utilized an *objective measure* as a primary endpoint. Survival at pre-specified endpoints (sodium phenylacetate and sodium benzoate combination [Ammonul], onasemnogene abeparvovec [Zolgensma]), and urinary free-cortisol concentration (pasireotide diaspartate [Signifor]) are some examples of the objective endpoints used. For the few cases where the endpoint was subjective, the benefit was so large it was unlikely to be due to chance alone. For example, in the case of burosumab (Crysvita, X-linked hypophosphatemia [XLH] in adult and pediatric patients ≥ 1 year, a rare disease), the studies in support of the pediatric indication used data from a retrospective natural history study conducted in 52 children who were on conventional therapies (phosphate/calcitriol) as external controls and a subjective clinician-reported outcome (reduction in total Rickets Severity Scale [RSS] scored by a radiologist) as the endpoint. The large effect size for reduction in RSS (50–59% versus 12% in the historical control^13^) supported the pediatric approval. Factors that strengthened this case were the assessment of radiographs (from both the retrospective natural history study and children treated with Crysvita) by the same blinded radiologist and the use of three propensity score analyses to mitigate several imbalances in the demographics (i.e., sex and baseline rickets scores) between the treatment and historical control groups. FDA’s statistical review noted that while the comparisons were imperfect they were still supportive of the conclusion that Crysvita is more effective than conventional therapies at correcting rickets in pediatric XLH. Ultimately, FDA determined that the unmet medical need and the totality of data, including improvements in secondary and pharmacodynamic endpoints, supported approval in the pediatric indication.

### Examples of Approvals Based on Different Types of External Controls

A relatively recent example of utilizing retrospective natural history data is FDA’s approval of Zolgensma (a gene replacement therapy) for infantile-onset spinal muscular atrophy (SMA) due to biallelic mutations, a rare disease with high unmet need. SMA is a serious, life-threatening disease where untreated patients will either die or require permanent ventilation by 24 months of age. Given the rare nature of the disease, data from 23 patients were successfully used as an external control. In this case, the natural history of SMA was predictable, the efficacy of Zolgensma was objectively measured, there was a large treatment effect (90% alive without ventilation versus 25% based on natural history), and there was evidence of a temporal association with the intervention.^14^

Another approval that provides interesting insights into the use of retrospectively collected natural history data is that of defibrotide sodium (Defitelio) approved for the treatment of adult and pediatric patients with hepatic veno-occlusive disease (VOD) after hematopoietic stem-cell transplantation (HSCT), a rare disease with an 80% mortality rate and no available treatment options. The primary endpoint in the Defitelio pivotal study (survival at day 100) was compared to historical control data selected by independent retrospective review of patient records. Supportive data came from a dose-finding study, a compassionate use study, and a registry study. The major review issues pertained to the selection of the historical control group. The patients included in the historical control group were selected by a blinded, independent medical review committee who screened subjects undergoing HSCT. The committee used narratives, inclusion/exclusion case report forms, and partially redacted medical charts to select patients to be included in the control group. Although data collection for the treatment and historical control groups spanned vastly different timeframes (2 years and 12 years, respectively), the inclusion and exclusion criteria were pre-specified and were similar for both groups. The number of subjects in the historical control group was reduced (in two rounds) from 6867 to 123 and finally to 32 patients who had developed VOD and received standard of care. The last round was conducted after an interim efficacy analysis raised some concerns about bias because the survival rate in the larger historical control group initially selected was substantially higher than the rate generally reported in the literature. To adjust for the confounding effect of the potential prognostic factors, propensity score adjusted analyses were performed using four pre-specified covariates (all baseline prognostic factors of survival). Nonetheless, the day 100 survival rates of treated patients (38 to 45%) were higher than the historical control group (25%), the supportive care arm of the registry (31%), and published literature (< 20%). While FDA’s review included comments regarding the small size of the chosen historical control group and the risk of Type I error given the unplanned interim analyses, FDA ultimately approved Defitelio based on the *totality and consistency of the data*, particularly the consistency of the survival results in the pivotal study and supportive studies.

An example of a case using baseline control data for regulatory decision making is deferiprone (Ferriprox), an oral therapy for transfusional iron overload due to thalassemia syndrome (a rare disease). Deferoxamine, the only available therapy^15^ at the time of Ferriprox’s new drug application (NDA) review, was not tolerated by all patients, leaving an unmet medical need. Initially, the sponsor received a complete response letter mainly due to uncertainty regarding the clinical meaningfulness of the change in a novel surrogate endpoint^16^ in a single pivotal study versus deferoxamine. Ultimately, an independent committee selected a subset of patients (in whom previous chelation therapy was inadequate) from the sponsor’s previously conducted clinical studies, to be included in a prospectively planned study. This study compared the selected patients’ pre- and post-Ferriprox treatment results and showed that treatment with Ferriprox significantly decreased serum ferritin in about 50% of refractory patients. The statistical reviewer noted “this study has several serious limitations including lack of randomization, lack of control group, high rate of missing data and ignoring the variation between studies by simply pooling, all of which can introduce biases to the primary outcome.” Nevertheless, FDA considered the use of a prospectively planned statistical analysis plan and the selection of patients by the independent committee allowed an adequate selection of patients for the trial, minimized the possibility of bias, and allowed for an adequate assessment of drug effect. The review documents noted “This trial can be considered an adequate and well-controlled trial under the CFR and ICH E10 guidance for regulatory purposes.” Ferriprox was approved under the accelerated approval regulations.

An unusual use of a historical control that leveraged data from previously conducted clinical studies, was the addition of a new indication (monotherapy in patients with partial seizures) for lamotrigine extended release tablets (Lamictal XR) which was reviewed at an advisory committee meeting.^17^ The supplemental NDA was based on a single study in which 223 patients who received one of two-dose levels were compared to a historical control group based on a retrospective analysis of control arms from eight studies previously conducted for other anti-epileptic products [18]. The sponsor considered use of placebo or pseudo-placebo controls unethical given the significant control data already available from previously conducted studies. At the advisory meeting, FDA presented a systematic evaluation of the key statistical issues based on the Pocock criteria [19], which were applicable to this situation, as the historical control data were specifically *derived from the control arm* from prior studies with similar designs and methods. This evaluation included the timeframe for assessment of seizure frequency and severity, how exit rate was calculated, medications at baseline, and regional differences between study and historical controls. The advisory committee agreed (14 yes/0 no) that the proposed historical control approach was acceptable in this specific circumstance. FDA’s presentations and discussions at the advisory committee demonstrate the importance of proactively assessing the comparability of an external control to the treatment group across multiple parameters and ensuring that endpoint evaluations and statistical methods address potential biases as thoroughly as possible. This precedent for use of historical controls from previously conducted clinical studies was later applied to other antiepileptic drugs, including lacosamide (Vimpat).

Finally, the recombinant antihemophilic factor Novoeight is an example where historical control data from nine publications were used as external controls to support its approval for prophylactic treatment of Hemophilia A, a rare disease. In this case, annualized bleeding rate (ABR) in patients treated prophylactically with Novoeight was compared with the ABR observed in historical controls treated with on-demand regimens. The historical ABR was calculated using data weighted by the number of patients in each of the nine published studies. Calculated mean ABR was 22 bleeds per patient per year for historical controls treated with on-demand regimens compared to 6.9 bleeds per patient per year in subjects treated with prophylactic Novoeight, a 68% reduction in bleeding rate for subjects treated with Novoeight prophylaxis as compared to on-demand therapy historical controls. This was considered acceptable for the approval of Novoeight for routine prophylaxis treatment.

### Statistical and Methodological Considerations When Using External Controls

It is outside the scope of this article to provide a comprehensive review of methodological and statistical topics pertaining to the use of external controls, but some important considerations are highlighted in this section.

**Fig. 4:**
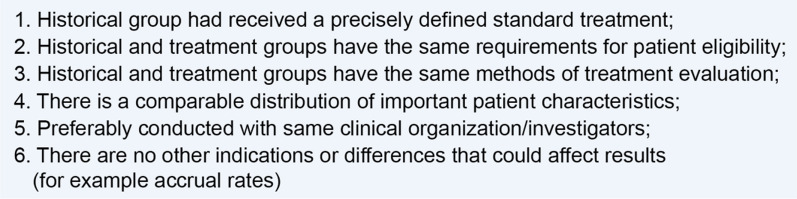
Pocock’s key criteria for accepting historical control data

A key challenge of using external controls is that differences in prognostic variables (such as demographics, diagnostic criteria, disease stage, baseline status, and concomitant therapies) between the treated and external control groups could lead to biases particularly in the absence of randomization. One way of addressing bias is through proper selection of the external control group. Pocock proposed six criteria for a historical control group to be acceptable [19] (Fig. 4), sometimes cited by FDA reviewers, as in the previously mentioned Lamictal XR example. However, Pocock specifically intended these criteria (deemed stringent by Lim et al. [20]) for specialized methods for combining a historical control group from a previous trial with a randomized concurrent control group. Indeed, Pocock’s use of the term “historical control” differs from his contemporaries [21, 22], and from current usage in reference to non-concurrent external controls in general (a historical control per ICH E10 guidance is any “well-documented population of patients observed at an earlier time”) [1]. Thus, while the Pocock criteria *may not all be applicable in a given situation*, those that are should be applied to the extent possible in the selection of a historical control group and to the ensuing comparative statistical analyses [23].

Three statistical methods are often used to adjust for baseline imbalances between the treated and external control groups: matching, covariate adjustment, and stratification. Propensity scores^18^ can be used as part of all three methods; one can match or stratify based on propensity score, or one could use a propensity score as a covariate [24, 25]. Propensity scores are a widely used and important method, but the method has its detractors. For example, Elze et al. [24] argue that propensity scores are not necessarily preferable to covariate adjustment, and King and Nielsen [26] raise fundamental questions about whether propensity scores succeed in addressing imbalance, inefficiency, model dependence, and bias.

Some variables may not fit into standard analysis approaches for covariates but may be addressed using other methods. An example is the contemporaneousness of external control versus trial data, which can be explored by examining the external control data for trends in outcome variables versus calendar date of assessment.

Bias can also be addressed by transparency about critical aspects of the analyses such as exclusion of subjects and handling of missing data. Missing data in particular represents a critical and widely studied issue. In brief, the principles of analyses to address missing data in nonrandomized trials “include the need to design and conduct trials to minimize the amount of missing data, the need to use principled missing data adjustments based on scientifically plausible assumptions, the need to conduct sensitivity analyses for potential deviations from the primary assumed mechanisms of missing data, and the need to collect covariate information that is predictive of missingness and the study outcomes” [27].

Another important set of issues pertains to longitudinal data. Longitudinal analysis approaches fully utilize the available data but present several challenges. For example, it may be difficult to align timepoints in the clinical trial with those of the external control because of the different assessment schedules or irregular assessment timing in the external data. Additionally, missing data may be a greater concern in a natural history data source than in a well-conducted clinical trial, given its observational nature as well as the potential for patients to enter and exit the database at various times, ages, disease states, etc. Cross-sectional analysis approaches, on the other hand, avoid complex methods for longitudinal missing data handling, but utilize less of the available data.

With all the considerations described above, an overarching principle is to apply alternative reasonable analysis approaches; consistency among the results lends confidence to the conclusions. When possible, consistency among results from different outcome measures, or multiple sources of external control data (sometimes preferable to attempting to combine the external control data sources), is valuable.

In the settings in which external controls are justified, it is critical to make optimal use of the available data, including retrospective natural history data, however imperfect. Statistical analysis should be carried out balancing practical matters with sound methodology.

## Discussion

Despite cautionary guidance from regulators, the use of external controls, including retrospective natural history data, to support FDA decision making is neither new nor particularly unusual, especially for orphan drugs. This review identified 45 cases in select therapeutic areas over the past 20 years where external controls were used in the pivotal trials supporting product approval. Nearly half (44%) of the 45 cases evaluated used controls sourced from retrospective natural history data; about one-third (33%) used controls sourced from patients’ baseline data; and the remainder used controls sourced from published data or previous clinical studies. Of note, none of the 45 cases where external controls were used were sourced from prospectively collected natural history data, perhaps not surprising knowing the difficulties of performing a meaningful prospective natural history study in a realistic time frame. This is a critical point given that the regulatory precedent is contrary to the FDA guidance which identifies prospective natural history as the gold standard, while discouraging use of retrospective natural history [3, 4]. While prospective natural history studies may be ideal, such an approach is often impractical, would lead to significant delays in the availability of life-saving therapies, and could ultimately stifle the development of products for rare diseases.

Recently, FDA has communicated that it is less swayed by the size of natural history studies than by the rigor of data collection and clarity on the course of the disease [28], and has recommended the use of longitudinal rather than cross-sectional data sources, as they yield more comprehensive information about disease onset and progression over time [3]. However, in a rare disease setting it is more likely that only cross-sectional data are available. Moreover, even when longitudinal data are available, longitudinal and cross-sectional statistical approaches both have potential advantages and should be used based on the objective of the statistical analysis.

External controls have been most frequently leveraged in situations where the conduct of prospective randomized, controlled studies was not feasible; examples include products approved for rare, life-threatening or severely debilitating conditions, in some cases slowly progressing, with no/inadequate available therapy. Good data quality and appropriate statistical analyses (including appropriate sensitivity analyses) are important factors in reducing bias when comparing new treatments to external controls. Furthermore, the use of common data standards such as CDISC (Clinical Data Interchange Standards Consortium) and OMOP (Observational Medical Outcomes Partnership) might facilitate standardization of data collection in observational studies and further minimize bias. Nevertheless, for small rare disease clinical trials, such comparisons cannot be perfect, and the ultimate decision should rely on the totality of evidence, recognizing that some unresolved questions may remain.

## Conclusion

Overall, the sponsors of the products identified appear to have achieved regulatory support to leverage external controls, including retrospective natural history, leading to successful product approval. Whilst acknowledging the limitations of the use of the external control, FDA invariably considered the nature and rarity of the condition, the unmet medical need, and ultimately the totality of evidence, including positive secondary or pharmacodynamic endpoints or positive data from supportive studies. Given this regulatory history, it is important to update FDA guidance to highlight the acceptability of retrospective natural history, to acknowledge the statistical challenges and provide recommendations for managing bias when using various types of external controls, and to facilitate information sharing. Such guidance would be a welcome addition to support clinical development and product approvals, especially in rare diseases.

## Footnotes

1 Statutory definition of “substantial evidence” is described in 21 USC 355(d)(7) as “evidence consisting of adequate and well-controlled investigations, including clinical investigations, by experts qualified by scientific training and experience to evaluate the effectiveness of the drug involved, on the basis of which it could fairly and responsibly be concluded by such experts that the drug will have the effect it purports or is represented to have under the conditions of use prescribed, recommended, or suggested in the labeling or proposed labeling thereof”.

2 21 Code of Federal Regulations (CFR) 314.126 describes the characteristics of an adequate and well-controlled study.

3 Regulation (21 CFR 314.126) uses “concurrent control” whereas ICH E10 also uses “internal control group” to describe a group of patients from the same population assigned to a different treatment.

4 Historical control (a type of external control) is also recognized by regulation 21 CFR 314.126 where “the results of treatment with the test drug are compared with experience historically derived from adequately documented natural history of the disease or condition, or from the results of active treatment, in comparable patients or populations.” This regulation notes “Because historical control populations usually cannot be as well assessed with respect to pertinent variables as can concurrent control populations, historical control designs are usually reserved for special circumstances. Examples include studies of diseases with high and predictable mortality (for example, certain malignancies) and studies in which the effect of the drug is self-evident (general anesthetics, drug metabolism).”

5 Note the International Conference of Harmonization (ICH) E10 guidance on “Choice of control groups and other related issues in clinical trials” uses the term *external control* while FDA Regulation (21 CFR 314.126) uses the term *historical control*, which is a subset of *external control*. According to ICH E10 guidance “An externally controlled trial compares a group of subjects receiving the test treatment with a group of patients external to the study, rather than to an internal control group consisting of patients from the same population assigned to a different treatment. The external control can be a group of patients treated at an earlier time (historical control) or a group treated during the same time period but in another setting”.

6 This includes both baseline-controlled studies and studies that use a sequential on–off–on (drug, placebo, drug) design, but that do not include a concurrently randomized control group.

7 A natural history study is an observational study intended to track the course of a disease over time with respect to demographic, genetic, environmental, and other variables (for example treatment modalities and concomitant medications) that correlate with the disease development and its outcomes. A natural history study may include patients receiving the current standard of care and/or emergent care and may be retrospectively or prospectively run.

8 A registry is an organized system that uses observational study methods to collect uniform data (clinical or other) to evaluate specific outcomes for a population defined by a particular disease, condition or exposure, and that serves one or more predetermined scientific, clinical or policy purposes. A registry may be used to collect disease information, recruit patients for clinical trials, monitor patient care and outcomes, advance research hypotheses, observe patient behavior patterns, establish disease-specific standards of care, or support reimbursement discussions. As such, they often play a vital role in the design of natural history studies.

9 RWE is clinical evidence about the usage and potential benefits or risks of a medical product derived from analysis of real-world data.

10 Unmet medical need is defined in FDA Guidance for “Expedited programs for serious conditions: drugs and biologics”.

11 Regulatory databases such as Cortellis, Pharmapendium, and the drugs@fda website were searched for review documents, approved labeling, and advisory committee meetings where some form of external control was mentioned.

12 Including three label expansions to add a new indication.

13 Only 30 of the 52 subjects in the historical control group had evaluable radiographic pairs and were included in the statistical analysis.

14 The clinical reviewer for Zolgensma noted “the natural history of infantile-onset SMA is well-documented and follows a relatively predictable course that can be objectively measured and verified; and the results of Study CL-101 indicated that the expected treatment effect is large, readily ascertained, and shows close temporal association with the intervention”.

15 Note although Exjade (deferasirox) was already accelerated approved for use as an iron chelator, consistent with guidance on “Available Therapy (July 2004)”, only Desferal (deferoxamine) was considered available therapy. However, not all patients tolerated Desferal due to administration difficulties.

16 Change in cardiac magnetic resonance imaging T2* parameter was a novel surrogate endpoint.

17 Lamictal XR supplemental NDA was discussed at the peripheral and central nervous system drug advisory committee meeting held on March 10, 2011.

18 A propensity score is defined as the estimated probability of assignment to a treatment group based on observed baseline covariates.
